# Global estimate of burnout among the public health workforce: a systematic review and meta-analysis

**DOI:** 10.1186/s12960-024-00917-w

**Published:** 2024-05-21

**Authors:** Ramya Nagarajan, Padmavathi Ramachandran, Rajendran Dilipkumar, Prabhdeep Kaur

**Affiliations:** 1 ICMR-National Institute of Epidemiology, Chennai, India; 2 Schizophrenia Research Foundation, Chennai, India; 3 Indian Institute of Sciences, Bengaluru, India

**Keywords:** Burnout, Public health personnel, Public health professional, Public health workforce, Exhaustion, Depersonalization, Personal accomplishment

## Abstract

**Introduction:**

Burnout is an occupational phenomenon resulting from chronic workplace stress. We conducted this review to estimate the pooled global prevalence of burnout among the public health workforce.

**Methods:**

We conducted this review as per the PRISMA 2020 guidelines. We included only cross-sectional studies reporting outcome estimates among the study population. We included articles published before December 2023. We used a search strategy to systematically select the articles from PubMed, Embase, and Google Scholar. We assessed the quality of the studies using an adapted version of NIH's study tool assessment for cross-sectional and observational cohort studies. We estimated the pooled proportion using the random-effects model.

**Results:**

We included eight studies in our review, covering a sample size of 215,787. The pooled proportion of burnout was 39% (95% CI: 25–53%; *p*-value: < 0.001). We also identified high heterogeneity among the included studies in our review (I^2^: 99.67%; *p*-value: < 0.001). Seven out of the eight studies were of good quality. The pooled proportion of the studies conducted during the COVID-19 pandemic was 42% (95% CI: 17–66%), whereas for the studies conducted during the non-pandemic period, it was 35% (95% CI: 10–60%).

**Conclusion:**

In our review, more than one-third of public health workers suffer from burnout, which adversely affects individuals' mental and physical health. Burnout among the public health workforce requires attention to improve the well-being of this group. Multisite studies using standardized definitions are needed for appropriate comparisons and a better understanding of variations in burnout in various subgroups based on sociodemographic characteristics and type of work responsibilities. We must design and implement workplace interventions to cope with burnout and increase well-being.

**Limitations:**

Due to the limited research on burnout among public health workers, we could not perform a subgroup analysis on various factors that could have contributed to burnout.

**Supplementary Information:**

The online version contains supplementary material available at 10.1186/s12960-024-00917-w.

## Introduction

The World Health Organization (WHO) defines burnout as an occupational phenomenon resulting from unsuccessfully managed chronic workplace stress [1]. It consists of three dimensions: exhaustion, cynicism related to one's job, and reduced professional efficacy [1]. Although burnout is not a medical diagnosis but a psychological phenomenon, it affects an individual's health under long-standing conditions [1–3]. Some common physical effects of burnout include hypercholesterolemia, type 2 diabetes mellitus, coronary heart disease, and musculoskeletal pain [4]. It also leads to job dissatisfaction and absenteeism in the workplace [4]. Burnout not only affects the professional status of an individual, but also plays an essential role in the development of various mental health issues [5]. Insomnia and depression are common mental health issues among individuals with burnout [4]. Burnout also exacerbates drug and alcohol abuse/dependency and suicidal ideation [6]. Even though the concept of burnout originated in the early 1970s, burnout is still widely prevalent due to existing environmental stressors and challenges individuals face at work [7].

Healthcare workers are a group of people involved in both direct and indirect patient care. As the patient load has increased in hospitals, burnout among the healthcare workers involved in direct patient care has risen in recent years [8]. The COVID-19 pandemic has further contributed to this rise in many ways [9]. Burnout research was initially focused on people-oriented occupations where a service provider directly deals with the recipient. The healthcare sector is people-oriented and has experienced increased burnout compared to the general population [10–12]. Even within the healthcare sector, burnout has been well-documented among doctors, nurses, and frontline workers. Abdo et al., Egypt 2015 reported moderate burnout among 66% of the physicians and nurses in a tertiary care hospital, while Youssef et al., Lebanon, reported moderate burnout among 90.7% of the study population [13, 14]. A study by Berger 2013 also revealed a threefold-fold greater incidence of burnout among emergency care physicians than general physicians in a hospital [15].

Burnout is a critical factor that leads to inefficiency and reduced productivity in health organizations [16]. Although there is extensive research on burnout among the healthcare workers involved in direct patient care, the evidence is limited to the public health workforce in managerial and administrative positions. Hence, we conducted this review to estimate the pooled proportion of burnout among the public health workforce, from grassroots-level community health workers to leadership-level policymakers.

## Methods

### Design and registration

We conducted this systematic review to estimate the global burden of burnout among the public health workforce. Before starting the data extraction process, we registered our review in PROSPERO (PROSPERO REG NO: CRD42022383238). We conducted our review per the Preferred Items for Systematic Reviews and Meta-Analyses (PRISMA) 2020 reporting guidelines [17].

### Eligibility criteria

*Study design* We included cross-sectional studies published in English in our review. We also included English-language literature documents that reported our outcome measure after satisfying the eligibility criteria. We excluded paid articles that we could not access. We searched for articles from the earliest record till December 2023.

*Study participants* We included studies conducted on the public health workforce. A public health workforce is a group of people working in public health departments in a country involved in administrative and managerial activities related to public health programs. This group of people varies from grassroots-level community health workers to leadership-level policymakers. We excluded studies conducted on health workers involved in direct patient care.

*Screening tool* We included studies reporting burnout using any valid tool. There are four validated tools available for measuring burnout [18]. The Maslach Burnout Inventory (MBI), a 22-item proprietary tool, was developed in 1981 to measure burnout among people-oriented professions [19]. However, as the MBI is a paid tool and concentrates more on people-oriented professions, its research use is limited among other groups [20]. Hence, in 1981, Dolan et al. developed a single-item measure for burnout, which is free and can be used in any occupational group [20]. However, Dolan et al. could not test the internal consistency reliability because it can be performed only on tools with three or more items [20]. In 2002, a 16-item Oldenburg Burnout Inventory (OBI) was developed in Germany [18]. It is free and can be applied to any occupational group [18]. In 2005, a 19-item Copenhagen Burnout Inventory (CBI) was designed to overcome some of the significant drawbacks of the MBI. The CBI was first used in the Project on Burnout, Motivation, and Job Satisfaction (PUMA) study in Denmark and was found to have satisfactory internal validity and reliability [21]. Recently, a fifth scale, the 23-item Burnout Assessment Tool (BAT), was developed by Schaufeli et al. 2020; this tool yields a single, composite burnout score [22]. However, the tool's optimum cutoff point for determining whether burnout was present or absent has yet to be determined [22].

*Outcome measures* We included studies reporting estimates of burnout among the study participants. We also had studies that reported various aspects of burnout, such as emotional exhaustion, depersonalization, and personal accomplishment. We included studies that adapted a validated tool to screen the outcome measure.

### Search sources and strategies

We conducted a systematic search of PubMed, Embase, and Google Scholar. To construct the search strategy, we retrieved MeSH, Emtree, and accessible search terms. We used search terms such as "public health professional", "public health workforce", "burnout", and "emotional exhaustion" to identify the articles. The detailed search strategy is given in supplementary Table 1. We also backreferenced the included studies to identify additional studies that could match our inclusion criteria.

### Study selection

*Primary screening* Two independent authors used Microsoft Excel to screen the title, abstract, and keywords (RN & PK). RN and PK retrieved the full texts of the eligible articles. The articles for which the full text was unavailable were excluded at this stage.

*Secondary screening* Two independent authors (RN & PK) conducted the full-text review to assess the eligibility criteria.

*Finalizing the study* A third author (PR) addressed disagreements during the screening process. All the authors agreed upon the final articles that were included in the study.

### Data extraction and management

Before starting the data extraction process, we prepared and piloted the data extraction sheet. The variables collected were study setting, study design, study participants, sample size, method of data collection, tool used for data collection, mean age, female population, and burnout measures. The investigator, RN, extracted the data, which another investigator, PK, cross-checked.

### Risk of bias assessment

We assessed the quality of the included studies using the adapted version of the National Institute of Health (NIH) study quality assessment tools for observational cohort and cross-sectional studies [23]. The original tool consists of 14 questions, including objectives, study participants, sample size, exposure variables, outcome variables, and analysis used in the study. Given that we included only cross-sectional studies in our review, we excluded questions exclusively designed for observational cohort studies (Q6, Q7, Q10, Q12, and Q13) from the tool. Hence, we considered only nine questions to assess the quality of the included studies. We awarded one point for each question addressed in the article. Thus, a maximum of nine points per article was allowed. We subsequently categorized the articles ranging from zero to four as "poor", five to six as "fair", and seven to nine as "good" [24].

### Statistical analysis

After systematically extracting the data, we used Stata version 16 (StataCorp, College Station, TX, USA) to analyze the data. We used the meta-analysis tab in Stata to analyze declaring the dataset as metadata [25]. We estimated the pooled effect size by proportion and 95% confidence interval (CI). We calculated weights for individual studies by the random-effects model with the DerSimonian and Laird method (dlaird) [26]. We conducted a Chi-square test of heterogeneity and the *I*^2^ statistic to quantify the between-study variance due to heterogeneity. We considered an *I*^2^ statistic < 25% mild, 25–75 moderate, and > 75% high heterogeneity [26]. We used a forest plot to represent the pooled estimate graphically. We also conducted a sensitivity analysis to assess the results' robustness and identify individual studies' influence on the overall pooled estimate [27]. We performed a subgroup analysis of the tools used for screening and the study period. We could not detect publication bias or meta-regression, as fewer than ten studies were included in our calculation [28, 29].

## Results

### Study selection

**Fig. 1:**
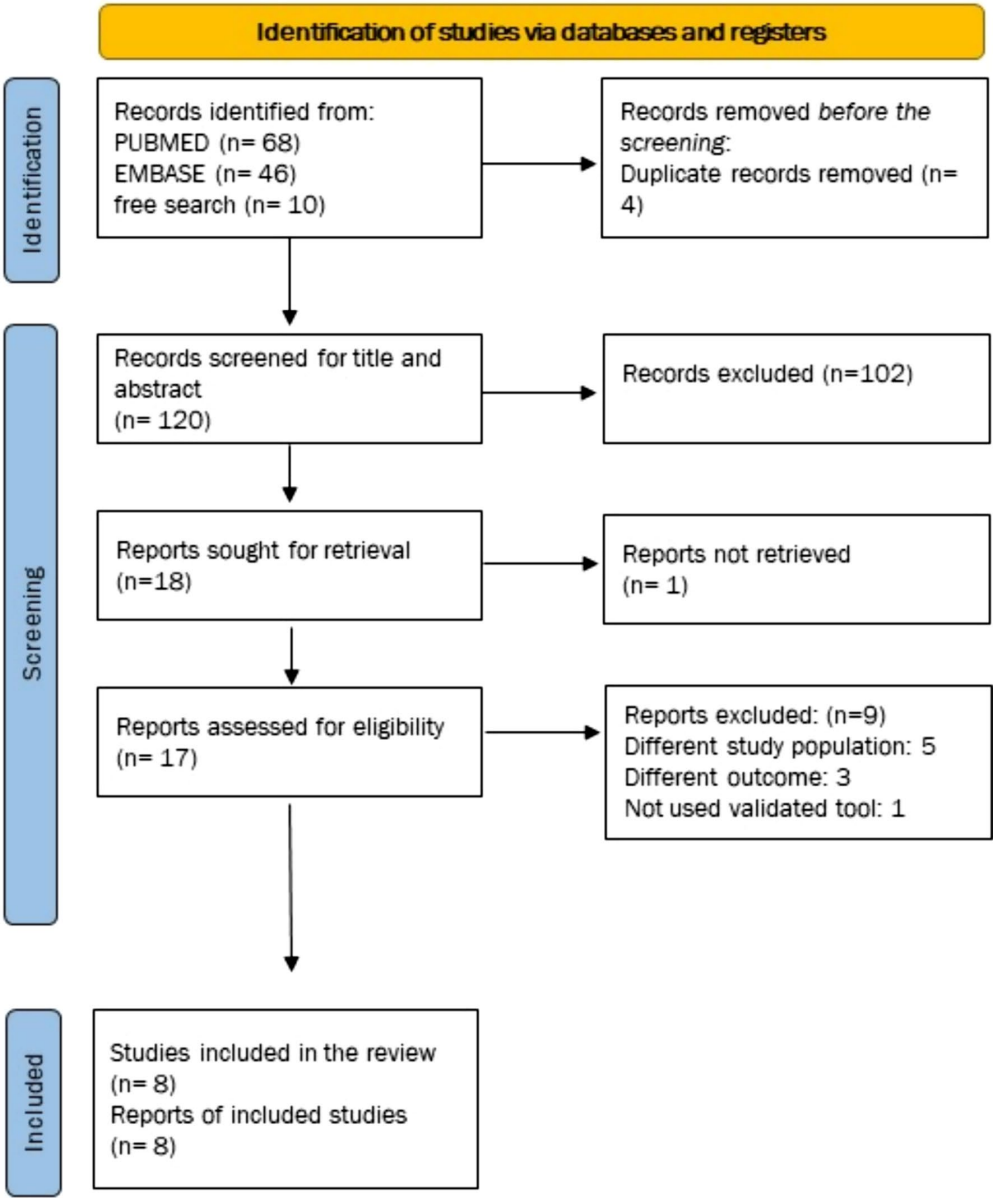
PRISMA 2020 flow diagram for new systematic reviews, which included searches of databases and registries only

After the primary screening, we identified 120 articles from the databases and records after excluding duplicates. We identified 68 articles from PubMed, 46 from Embase, and 10 from Google Scholar. We excluded 102 articles after the title, abstract, and keyword search. We selected 18 articles for full-text review. We excluded one article (Rossi et al. 2012) at this stage due to restricted access to the full text [30]. We excluded Rossi et al. (2012) as we could not get adequate data on burnout from the content provided in the abstract [30]. Finally, out of the 17 retrieved articles, five, three, and one were excluded because the study population, outcome measure, and use of nonvalidated tools did not match our eligibility criteria. Thus, eight articles were included in our systematic review and meta-analysis (Fig. 1). We also reported our review per the PRISMA 2020 guidelines in Supplementary Table 2.

### Characteristics of the included studies

The characteristics of the eight included studies are described in Table 1 [31–38]. *Sample size:* The median sample size of the included studies is 313.5 (IQR: 157–11,885). The smallest sample size was 27, and the largest was 104,928. The total sample size of all the included studies was 215,787. *Study setting*: The review covers studies conducted in different economic settings. Of the eight studies, one is a multicountry study including 14 countries across southeast Asia and western Pacific regions, four from high-income countries (USA, South Korea, and Japan), two from upper-middle-income countries (China and Malaysia), and one from Lower-middle income country (India). *Study period*: Two of the eight studies were conducted in 2019, one in 2020, two in 2021, and three in 2022. Of these eight studies, five were conducted during the COVID-19 pandemic, while three were conducted during non COVID-19 pandemic period. *Study participants and design*: All the included studies were performed on the public health workforce following a cross-sectional study design. *Screening tool*: All the studies included used a validated tool to measure burnout. Nearly four studies have used the MBI to screen burnout among the study population. Only two and one studies applied OBI and CBI, respectively. *Outcome measure*: Of the eight studies, all presented an overall estimate of burnout. In addition, three studies have also given estimates for emotional exhaustion, depersonalization, and personal accomplishment. *Risk of bias assessment*: Seven articles were of good quality based on the adapted version of the NIH's study quality assessment tools for observational cohort and cross-sectional studies (Supplementary Table 3).

**Table 1:** Characteristics of the included studies (*N* = 8)

Study	Study setting	Study design	Diagnostic tool and survey mode	Study participants	Sample size	Mean age (years)	Female population	Outcome measure
Stone 2021	USA*	Cross-sectional study	Single Item Burnout Measure; Online Survey	Public health professionals (overall)	225	47	185	Burnout: 66.2%
				Individuals in public health practice	176	NA	NA	Burnout: 65.1%
				Individuals in public health academics	27	NA	NA	Burnout: 85.2%
Jang 2021	South Korea	Cross-sectional study	MBI^†^; Online Survey	Public health officers	261	35	NA	Burnout: 60.2 EE^‡^: Mean (SD): 31.2 (13.5); high EE^‡^: 60.2%
Nishimura 2022	Japan	Cross-sectional study	MBI^†^; Online Survey	Public health officers	100	NA	80	Burnout: 27% EE^‡^: Median (IQR): 17 (8.0–26.8) DP^§^: Median (IQR): 4 (1.0–6.8) PA**: Median (IQR): 24 (12–32)
Ryu 2019	Southeast Asia, Western Pacific	Cross-sectional study	MBI^†^-Health Services Survey; Offline survey	Field epidemiologist	62	NA	NA	Burnout: 19% EE^‡^: high: 24.2%; moderate: 63%; low: 13% DP^§^: high: 34%; moderate: 58%; low: 8% PA**: high: 23%; moderate: 71%; low: 6%
Yeager 2019	USA	Cross-sectional study	OLBI^††^; Online Survey	Public health workforce (overall)	104,928	NA	NA	Burnout: 26.45%
				Individuals in the local health department	70,302	NA	NA	Burnout: 26.04%
				Individuals in state health agencies	34,626	NA	NA	Burnout: 27.3%
Ibrahim 2022	Malaysia	Cross-sectional study	OLBI^††^; Online Survey	Public health workforce	366	35.5	269	Burnout: 44.5% Disengagement: Mean (SD): 2.43 (0.23) Exhaustion: Mean (SD): 2.13 (0.48)
Lu 2020	China	Cross-sectional study	MBI^†^-General Survey; Online survey	Public health service providers	4304	35.3	3231	Burnout: 58.06%; Mean (SD): 1.77 (1.02) EE^‡^: Mean (SD): 2.15 (1.34); high EE^‡^: 19.19% DP^§^: Mean (SD): 1.21 (1.19); high DP^§^: 18.12% PA**: Mean (SD): 4.19 (1.38); low PA**: 47%
Yella 2022	India	Cross-sectional study	CBI^‡‡^; Offline Survey	Community health workers	410	34	410	Burnout: 10.5%; Mean (SD): 34.3 (15.1)

*United States of America

^†^Maslach Burnout Inventory

‡Emotional exhaustion

^§^Depersonalization

**Personal accomplishment

††Oldenburg Burnout Inventory

^‡‡^Copenhagen Burnout Inventory

### Pooled estimation of burnout among public health workers

**Fig. 2:**
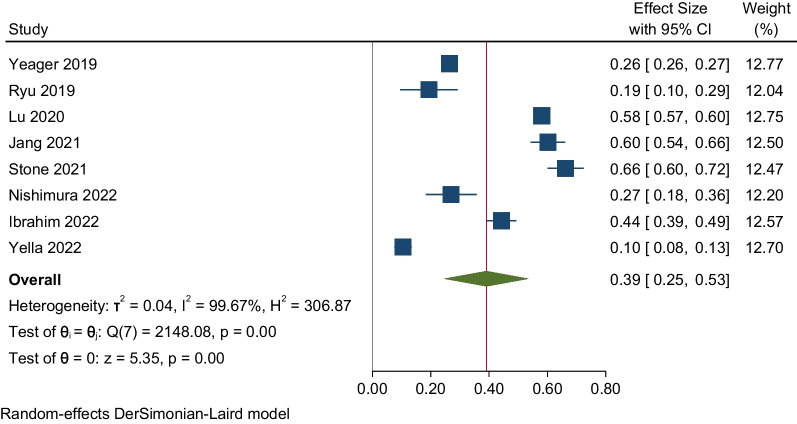
A graphical representation of the pooled estimate of burnout among the public health workforce: Forest plot (*N* = 8)

*Global prevalence* The proportion of burnout in the included studies ranged from 10.5 to 85.2% (Table 1). We estimated the pooled proportion of burnout among the public health workforce and graphically represented it using the forest plot in Fig. 2. The pooled proportion of burnout was 39% (95% CI: 25–53%; *p*-value: < 0.001). This shows that 39% of the global public health workforce has reported burnout. We also identified high heterogeneity among the included studies in our review (I^2^: 99.67%; *p*-value: < 0.001).

### Subgroup analysis

*Prevalence by screening tool used* We conducted a subgroup analysis to estimate the pooled prevalence of burnout by the tool used for screening. Studies using the Maslach Burnout Inventory had a pooled prevalence of 42% (95% CI: 25–58%), and those using the Oldenburg Burnout Inventory had a pooled prevalence of 35% (95% CI: 18–53%) (Supplementary Fig. 1). *COVID-19 and burnout*: The pooled proportion of the studies conducted during the COVID-19 pandemic was 42% (95% CI: 17–66%), whereas for the studies conducted during the non-pandemic period, it was 35% (95% CI: 10–60%) (Supplementary Fig. 2).

### Publication bias, meta-regression, and sensitivity analysis

We could not assess publication bias or meta-regression, as only eight studies were included in our review [39]. However, we performed a sensitivity analysis and found none of the studies influenced the overall pooled estimate (Supplementary Fig. 3).

## Discussion

Burnout is an occupational phenomenon and is advised not to apply to other aspects of life [1]. More than one-third of the global public health workforce has reported burnout. Studies using the MBI for screening have reported a higher pooled incidence than those using other tools, and studies conducted during the COVID-19 pandemic have reported a greater pooled incidence of burnout than those conducted during non-pandemic times.

*Global perspective on burnout among public health workforce* The overall burnout risk based on our review ranged from 10.5 to 66.2%, comparable to that of other health professionals. A systematic review by Rotenstein et al. (2018) reported that overall burnout among physicians varies from 0 to 80.5%, while another study by Karuna et al. (2022) noted that the estimates range from 6 to 33% [40, 41]. The data suggest that burnout is a major issue in the public health workforce.

*Addressing burnout in the public health workforce* The public health department is the backbone of the health system in a country. The public health workforce is involved in assessing the extent of public health problems, formulating programs and policies to prevent and control diseases, and executing control strategies at the community level. They lead outbreaks and emergency responses and work with multiple stakeholders. There are limited data about the extent of burnout in various settings. One such initiative is the Public Health Workforce Interests and Needs Survey, which was conducted in 2014, 2017, and 2021 by the de Beaumont Foundation and the Association of State and Territorial Health Officials, USA, to document the strengths and needs of public health workers [42]. In 2017, burnout was reported in 26.45% of public health workers, while in 2021, 56% of public health workers reported at least one symptom of posttraumatic stress disorder [32, 43]. We recommend conducting similar surveys in other countries to capture public health workers' mental health and needs. Unless we generate adequate evidence regarding the burden of the problem, it will be impossible to design interventions and advocate for policymakers to allocate resources to address their needs.

*Variability in burnout assessment tools* The burden of burnout varies depending on the tools used in various studies. A study using the CBI reported a prevalence of 10%, while a single-item burnout measure reported a prevalence of 66.2%. The results of the studies using the MBI and OBI varied from 19–60% and 26–44%, respectively. There is a considerable difference in reporting symptoms according to each tool. The MBI and CBI summarize symptoms on three subscales, whereas the OBI and single-item burnout measure summarize symptoms on two and one subscales, respectively. The studies did not explicitly give the cutoff for each scale for classifying burnout as present or absent. Hence, our pooled estimate of burnout must be interpreted cautiously. Although pooling studies based on the tool used for screening burnout would be the best strategy, we could not perform a subgroup analysis based on all the available instruments due to the limited number of studies.

*Impact of the COVID-19 pandemic on burnout* The COVID-19 pandemic has significantly impacted everyone's physical and mental health [44–47]. According to our review, the pooled estimates of burnout in the studies conducted during the COVID-19 pandemic were greater than those reported during the non-COVID-19 pandemic. These results were similar to those of Ulbrichtova et al. 2022 and Lasalvia et al. 2021, who reported that all three subscale scores were greater in the COVID-19 group than in the non-COVID-19 group [48, 49]. Although all professional groups faced hardships during the COVID-19 pandemic, the public health workforce was involved in multiple response and control activities under difficult working conditions. The patients were responsible for community care of the asymptomatic patients, contact tracing, quarantine and isolation activities, data entry, informed decisions, and policymaking. Like frontline workers, public health workers are massively short staffed and do not work on a shift basis, significantly impacting their burnout [50].

*Strengths and limitation* The major strength of our review was the unique nature of the study population, for which there is limited data. Our review must be interpreted cautiously after considering the following limitations. First, we included only published open-access literature in peer-reviewed journals in English. Thus, we could miss the information in the grey literature, closed-access journals, conference proceedings, and government portals. Second, we could not assess publication bias or perform meta-regression due to our review's limited number of studies. Hence, we could not establish the presence or absence of publication bias or potential variables contributing to heterogeneity in our review. Third, as there was limited evidence on burnout by age, sex, and years of experience, we could not perform a subgroup analysis based on these factors. Thus, we recommend conducting further research to identify additional information on these aspects.

## Conclusion

To conclude, burnout among the public health workforce requires attention to improve the well-being of this group. The COVID-19 pandemic has brought this to the limelight. As a further extension to this review, we have planned to conduct a mixed-method study to estimate the prevalence of psychological distress and burnout and to explore the challenges and facilitators experienced by public health managers in the country. We also recommend multisite studies using standardized definitions, which enables appropriate comparisons and a better understanding of variations in burnout in various subgroups based on sociodemographic characteristics and type of work responsibilities.

### Supplementary Information

**Additional file 1.** Supplementary tables and figures.

## Data Availability

The data are available from the corresponding author. The material will be made available upon request to the corresponding author.
